# High-Resolution Tandem Mass Spectrometry Identifies a Particular Ganglioside Pattern in Early Diabetic Kidney Disease of Type 2 Diabetes Mellitus Patients

**DOI:** 10.3390/molecules27092679

**Published:** 2022-04-21

**Authors:** Anca Suteanu-Simulescu, Alina Diana Zamfir, Raluca Ica, Mirela Sarbu, Cristian V. A. Munteanu, Florica Gadalean, Adrian Vlad, Flaviu Bob, Dragos Catalin Jianu, Ligia Petrica

**Affiliations:** 1 Department of Internal Medicine II, Division of Nephrology, “Victor Babes” University of Medicine and Pharmacy, 300041 Timisoara, Romania; anca.simulescu@yahoo.com (A.S.-S.); flaviu_bob@yahoo.com (F.B.); ligia_petrica@yahoo.co.uk (L.P.); 2 Department of Nephrology, County Emergency Hospital, 300723 Timisoara, Romania; 3 Centre for Molecular Research in Nephrology and Vascular Disease, Faculty of Medicine, “Victor Babeș” University of Medicine and Pharmacy, 300041 Timisoara, Romania; vlad.adrian@umft.ro (A.V.); dcjianu@yahoo.com (D.C.J.); 4 Department of Condensed Matter, National Institute for Research and Development in Electrochemistry and Condensed Matter, 300569 Timisoara, Romania; alina.zamfir@uav.ro (A.D.Z.); raluca.ica@gmail.com (R.I.); mirela.sarbu86@yahoo.co.uk (M.S.); 5 Department of Technical and Natural Sciences, “Aurel Vlaicu” University of Arad, 310330 Arad, Romania; 6 Department of Physics, West University of Timisoara, 300223 Timisoara, Romania; 7 Department of Bioinformatics & Structural Biochemistry, Institute of Biochemistry, 060031 Bucharest, Romania; cristian.v.a.munteanu@gmail.com; 8 Department of Internal Medicine II, Division of Diabetes and Metabolic Diseases, “Victor Babes” University of Medicine and Pharmacy, 300041 Timisoara, Romania; 9 Department of Diabetes and Metabolic Diseases, County Emergency Hospital, 300723 Timisoara, Romania; 10 Department of Neurosciences, Division of Neurology, “Victor Babes” University of Medicine and Pharmacy, 300041 Timisoara, Romania; 11 Centre for Cognitive Research in Neuropsychiatric Pathology (NeuroPsy-Cog), Department of Neurosciences, “Victor Babes” University of Medicine and Pharmacy, 300041 Timisoara, Romania; 12 First Department of Neurology, County Emergency Hospital, 300723 Timisoara, Romania

**Keywords:** nanoelectrospray, high-resolution tandem mass spectrometry, screening, fragmentation analysis, diabetic kidney disease, ganglioside biomarkers

## Abstract

Considering the valuable information provided by glycosphingolipids as molecular markers and the limited data available for their detection and characterization in patients suffering from Type 2 diabetic kidney disease (DKD), we developed and implemented a superior method based on high-resolution (HR) mass spectrometry (MS) and tandem MS (MS/MS) for the determination of gangliosides in the urine of DKD patients. This study was focused on: (i) testing of the HR MS and MS/MS feasibility and performances in mapping and sequencing of renal gangliosides in Type 2 DM patients; (ii) determination of the changes in the urine gangliosidome of DKD patients in different stages of the disease—normo-, micro-, and macroalbuminuria—in a comparative assay with healthy controls. Due to the high resolution and mass accuracy, the comparative MS screening revealed that the sialylation status of the ganglioside components; their modification by *O*-acetyl, CH_3_COO^−^, *O*-fucosyl, and *O*-GalNAc; as well as the composition of the ceramide represent possible markers for early DKD detection, the assessment of disease progression, and follow-up treatment. Moreover, structural investigation by MS/MS demonstrated that GQ1d(d18:1/18:0), GT1α(d18:1/18:0) and GT1b(d18:1/18:0) isomers are associated with macroalbuminuria, meriting further investigation in relation to their role in DKD.

## 1. Introduction

Type 2 diabetes mellitus (DM) remains a therapeutic challenge with a constantly increasing global prevalence, despite scientific advances in the field. Associated with devastating macrovascular and microvascular long-term damage, Type 2 DM leads to increased mortality.

Diabetic kidney disease (DKD) is the most common cause of end-stage renal disease (ESRD) in developed and developing countries [1,2]. Thus, early detection of DKD and optimal management of Type 2 DM are crucial in order to reduce complications, morbidity, and mortality.

Currently, the diagnosis and progression of DKD relies on the measurement of albuminuria and the steady reduction in glomerular filtration rate (eGFR), both parameters showing modest prediction of future renal status. The natural history of DKD includes several clinical stages, including the following: early glomerular hyperfiltration, the occurrence of microalbuminuria or macroalbuminuria, a decline of glomerular filtration rate, and ESRD. However, recent research considers albuminuria to be a marker of deteriorating condition [3]. In the last decade, particular interest has been shown in the tubulocentric concept, according to which the proximal tubule and the tubulointerstitial compartment could have an essential role in the initiation and progression of DKD [4,5,6].

In this context, the development of prognostic and predictive glycomic biomarkers is gaining increasing attention and has become the focus of many research groups over the years [7,8,9,10,11].

Gangliosides represent a particular class of glycosphingolipids with a complex structure identified in all tissues and body fluids and particularly abundant in the central nervous system (CNS) [12]. As essential constituents of the neuronal cell bilayer membranes, gangliosides play a vital role in brain development, maturation and aging, mediating cell adhesion, activation, proliferation, motility, and growth. Gangliosides consist of a ceramide of different composition with respect to the sphingoid base, fatty acid residues rooted into the outer layer of the membrane and a sialylated oligosaccharide chain covering the membrane and participating in specific and essential interactions with other molecules in the surrounding environment. Through normal cell surface turnover, known as “cell surface shedding”, gangliosides are released, to a certain extent, into the intercellular space.

Because the expression, distribution, and structure of gangliosides are known to be cell and tissue type-specific and to vary during tissue development, maturation, aging, and most of all, in pathological states [12], gangliosides are considered among the most valuable diagnostic markers and prospective therapeutic agents.

Several methods using serum or kidney biopsy fragments have been applied for renal ganglioside identification in Type 2 DM patients, such as thin-layer immunostaining [7,8], high-performance thin-layer chromatography (HPTLC) [9], spectrophotometry [10], and mass spectrometry (MS) [11].

In view of the valuable information that can be provided by gangliosides as molecular markers of neuronal tissue [12] and the limited data available so far in detection and characterization of glycosphingolipids in the urine of DKD patients, we developed and implemented a superior method based on nanoelectrospray (nanoESI) high-resolution (HR) MS for the determination of gangliosides in the urine of DM patients. This option for HR MS was guided by the advantages offered by this method in the analysis of the complex mixtures of gangliosides extracted from human urine. The elevated resolving power of the instrument: (i) allows for the detection of ions of close *m*/*z* values, which otherwise could not be discriminated in a heterogeneous multicomponent sample containing species of various lengths and architectures of the glycan chain and dissimilar compositions of the ceramide; (ii) basically eliminates the need for mixture separation by either liquid chromatographic or electrophoretic methods prior to MS analysis; (iii) provides a high mass accuracy, which greatly increases the reliability of structural identification; and (iv) offers not only a better insight into the complexity of DM-associated gangliosidome but also data on the species with potential biomarker role based on the high confidence in mass determination for both molecular and fragment ions.

This pilot study, with the aim of characterizing urine gangliosides with biomarker roles in the early diagnosis of DKD, was focused on: (a) testing the feasibility and performance of HR MS and tandem MS (MS/MS) by high-energy collision-induced dissociation (HCD) in mapping renal gangliosides in Type 2 DM patients; (b) determination of the changes in the expression of native ganglioside mixtures from the urine samples of Type 2 DM patients in a comparative assay with healthy controls and characterization of the discovered biomarkers. For this purpose, we optimized a modern bioanalytical platform based on nanoESI HR MS on an Orbitrap instrument tuned for operation in negative ion mode in both MS screening and MS/MS fragmentation for detailed structural analysis.

## 2. Results and Discussions

### 2.1. Comparative Screening of the Samples by nanoESI HR MS

The A1, A2, A3, and C ganglioside samples were infused by nanoESI one after the other into an Orbitrap MS and screened in negative ion mode under identical instrumental conditions. In each case, the total ion current (TIC) signal was acquired for 2 min, generating spectra from the accumulation of 100 scans. Due to the high sensitivity of the method, even under the employed time-restrictive conditions, the acquired mass spectra featured a high *signal-to-noise* ratio and a rich molecular ion pattern.

Assessment of the mapping data generated under the same conditions revealed significant differences in the number and type of ganglioside components expressed in the A1, A3, and C samples, whereas no differences between the A1 and A2 samples were observed. These results are summarized in Table 1, which comparatively lists the structures identified in the A1, A3, and C native mixtures, together with the experimental *m*/*z*_exp_ values of the detected signals and the theoretical *m*/*z*_theor_ values corresponding to the proposed structures.

The high-resolution and mass accuracy of the employed MS platform enhanced the discrimination and identification based on accurate mass measurements of no less than 37 distinct urine ganglioside and fucoganglioside components in the three samples. The discovered species belong to 15 different classes, including the modifications of the main glycan chain (Table 2), and were assigned the excellent average mass accuracy of 4 ppm.

A detailed evaluation of the structures in Table 1 revealed that out of all samples, the A3 mixture, which corresponds to macroalbuminuria (hence, to an advanced stage of DKD), contains the highest number of distinct ganglioside compounds, i.e.,19, followed by sample C, with 12 distinct species, and sample A1, with 10.

**Figure 1:**
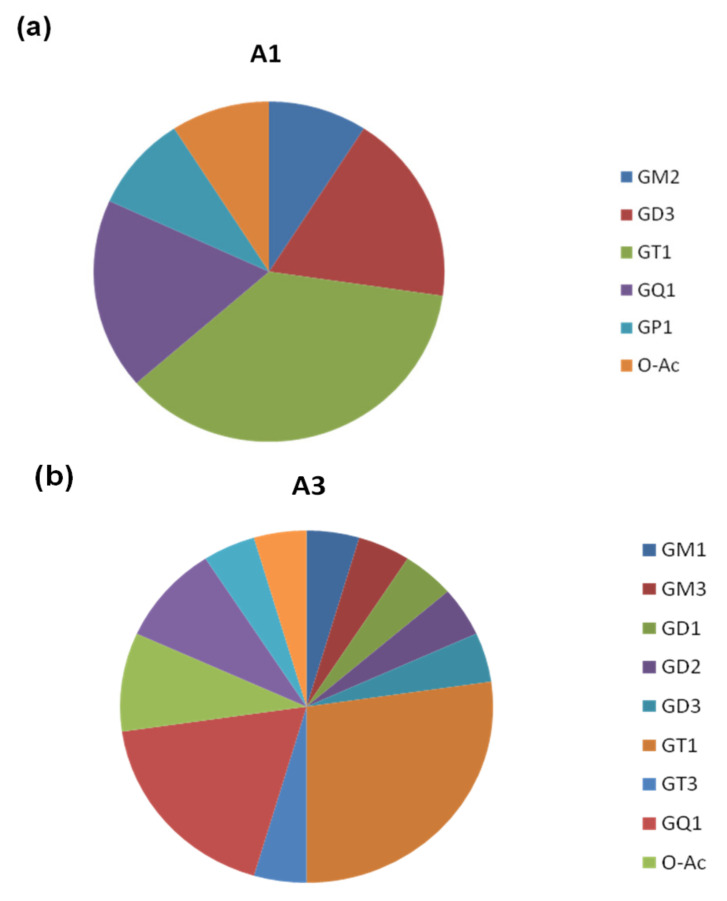

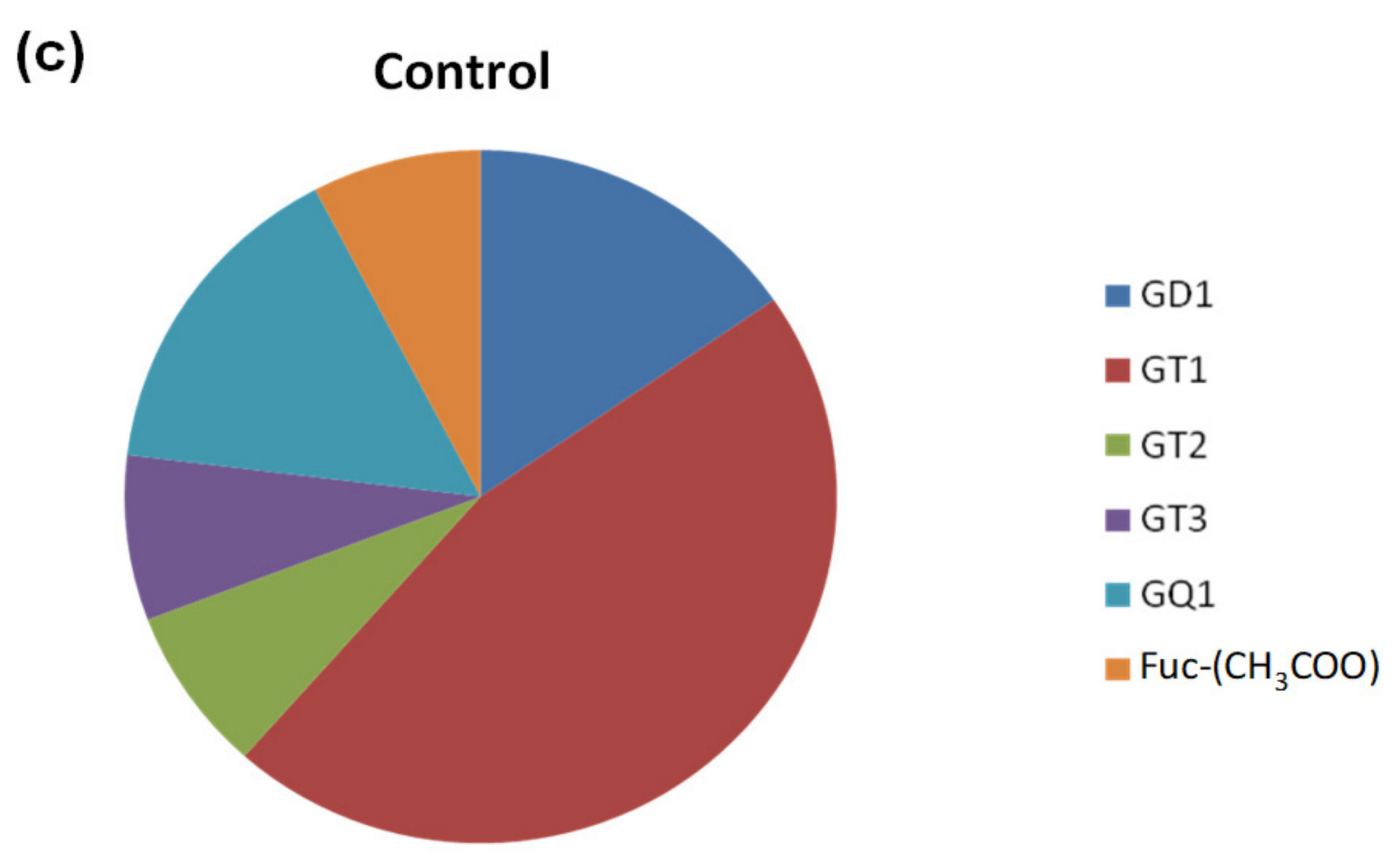
Pie charts representing the ganglioside classes and their proportions in (**a**) A1, (**b**) A3, and (**c**) C samples.

**Figure 2:**
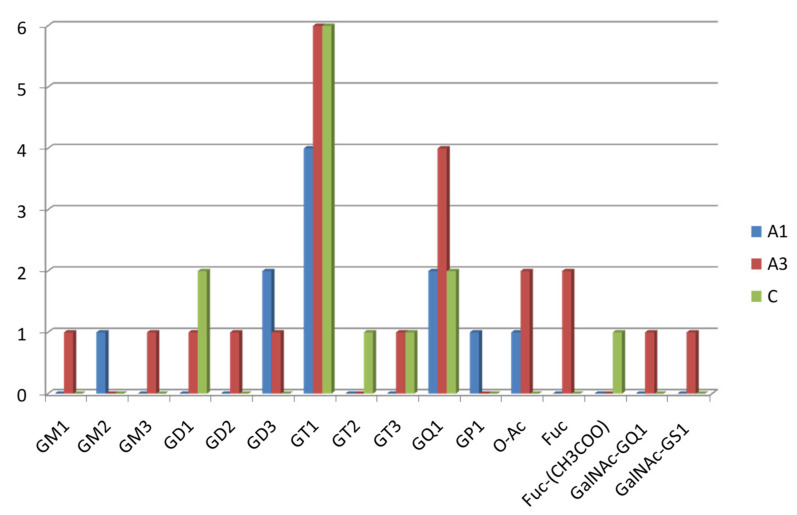
Histogram comparatively plotting the number of ganglioside species in A1, A3, and C vs. their glycan chain composition.

As compared to the A1 and C samples, A3 also: (i) encompasses the highest number of species with the longest *O*-glycan chain belonging to the G1 class and the most prominent diversity of ceramide compositions; (ii) presents the highest number of ganglioside classes expressed—12 classes vs. only 6 in the A1 sample and 6 in the C sample, respectively; (iii) contains the highest number of gangliosides, which exhibit a saccharide core altered by non-carbohydrate *O*-acetyl, as well as carbohydrate *O*-fucosyl and *O*-GalNAc biologically relevant modifications; (iv) is the only urinary extract containing ganglioside structures modified by *O*-GalNAc attachment, namely GalNAc-GS1(t18:1/18:0), detected as a quadruply deprotonated and sodiated molecule at *m*/*z* 1176.8266, and GalNAc-GQ1(d18:1/18:0), as a doubly deprotonated molecule at *m*/*z* 1310.1271. As is visible in Figure 1a–c, showing the proportion of the ganglioside classes expressed in the three samples, and in the comparative histogram depicted in Figure 2, A3 extract displays a particular sialylation status, being dominated by polysialocompounds. Except for the monosialylated gangliotetraose GM1(d18:1/16:1) detected as [M-2H^+^]^2^^−^ at *m*/*z* 756.9078 and the monosialylated GM3(d18:0/24:0) detected as a doubly deprotonated molecule at *m*/*z* 645.3956, all 17 other species contain more than one Neu5Ac residue in their glycan moiety. Moreover, because no less than seven trisialo GT1, four pentasialo GQ1, and even a heptasialylated GS1 were discovered, obviously, next to the augmented number and diversity of structures, the length of the glycan cores, and their peripheral modifications, the high overall sialylation content represents another feature specific to macroalbuminuria.

A clinically relevant observation also emerges from the A1 vs. C comparative assay. Whereas in the control sample, C, the highest sialylation degree of the detected gangliosides is four, identified via two tetrasialylated gangliotetraose GQ1 differing in the composition of their lipidic aglycone, in the A1 sample, the highest sialylation degree is four. Next to the di-, tri-, and tetrasialylated gangliosides, the A1 mixture contains a pentasialo gangliotetraose GP1(d18:1/18:0), detected as [M-4H^+^]^4−^ ion at *m*/*z* 676.5569, which was assigned the excellent mass accuracy of 2.95 ppm. Such data related to the sialylation status of the investigated samples show that the sialylation degree of renal gangliosides follows the trait C<A1<A2=A3, an observation that emphasizes the precocity of these modifications, even in the normoalbuminuria stage of Type 2 DM patients. Evidently, the sialylation degree increases with the progression of the disease, which makes it a molecular parameter to be considered not only for early detection of DKD but also for the assessment of DKD progression and treatment effectiveness.

A detailed inspection of the aglycone constitution reveals that, except for the marked dissimilarities in the composition of the glycan chain, the gangliosides expressed in the three samples also exhibit differences in the structure of their ceramides. The most evident alterations of the lipid part are related to the less common process of sphingoid base trihydroxylation, which occurs in the A1 and A3 ganglioside mixtures, and the unusual length of the fatty acid chains, present solely in A3. Each of the A1 and A3 samples was found to contain two species with trihydroxylated sphingoid bases of the ceramide. Hence, GT1(t18:0/18:0) and GT1(t18:0/20:0) were discovered in the A1 sample, whereas the more complex trihydroxylated GT1(t18:1/24:3) and Fuc-GT3(t18:1/18:3) structures were found in the A3 sample.

Remarkably, the A3 sample also shows evidence of species containing verylong-chain fatty acids (VLCFA). A VLCFA is considered a rarely occurring lipid structure encompassing 23 to 27 carbon atoms in the fatty acid chain. Four such unusual VLCFA species carrying 24 carbon atoms in the fatty acid chain were detected in the A3 sample: GM3(d18:0/24:0), GT1(t18:1/24:3), *O*-Ac GT1(d18:0/24:0), and Fuc-GT1(d18:0/24:0), of which one structure also features the uncommon sphingoid base trihydroxylation and three double bonds in the fatty acid chain, whereas two structures present attachments to the glycan core of *O*-Ac and fucosyl group, respectively.

These characteristics of the ceramide portions in the A1 and A3 samples, which were not found in the control sample; the similarities between the A1 and A2; and the marked differences detected in the A3 suggest that, next to the carbohydrate composition and the sialylation status, the structure of the lipid moiety in renal gangliosides is another possible marker of early DKD. Moreover, the modifications in the ceramide composition appear to depend on the albuminuria stages; complex ceramides, exhibiting trihydroxylation of the sphingoid base and VLCFA seem to be linked to the advanced albuminuria stages. Considering that the uncommon number of three double bonds in the fatty acid chain was encountered only in the A3 sample via two different species, Fuc-GT3(t18:1/18:3) and GT1(t18:1/24:3), seemingly, the mechanism of double bond formation is also related to the progression of DKD.

The negative effects exerted by lipid accumulation within the kidney, at both the glomerular and tubular level, are cell-specific. Moreover, lipotoxicity is associated with various types of lipids, among which gangliosides display a central-stage role [13].

The kidney content of gangliosides, among other lipid classes, was increased within podocytes, as well as proximal tubule cells, in a DN mouse model [14]. Proximal tubules may be exposed to high levels of urinary lipids, including gangliosides, due to increased fatty acid content per albumin molecule early in DKD [15]. The direct consequences of lipid accumulation within the segments of the nephron are related to structural and functional modifications, which lead to impaired albumin processing early in the course of Type 2 DM.

The vast majority of studies that focus on the characterization of renal gangliosidome rely on experimental models of diabetic nephropathy. To the best of our knowledge, this is the first human translational study from basic research to clinical applicability to report on a particular urinary ganglioside pattern in patients with Type 2 DM. The practical significance of our study is in the demonstration of an association of the sialylation degree of urinary gangliosides and the structure of their ceramides with early DKD, staged by albuminuria level.

### 2.2. Detailed Structural Analysis of Polysialylated Species Associated to Macroalbuminuria by HCD MS/MS

**Figure 3:**
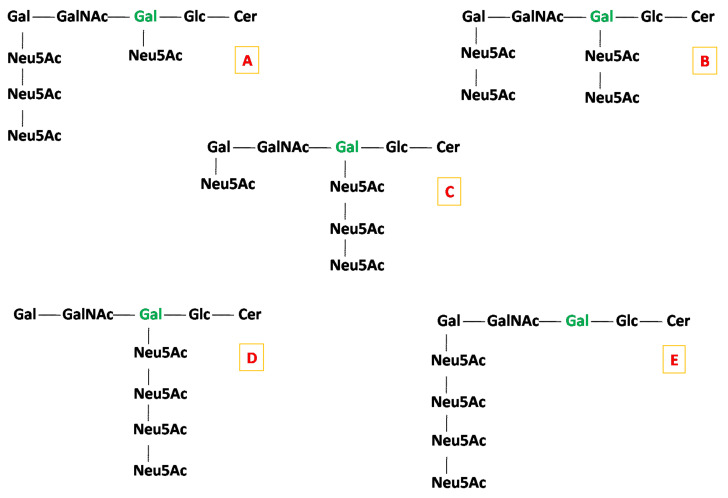
The five most common isomers of GQ1(d18:1/18:0): (**A**) GQ1a(d18:1/18:0) isomer; (**B**) GQ1b(d18:1/18:0) isomer; (**C**) GQ1c(d18:1/18:0)isomer; (**D**) GQ1d(d18:1/18:0) isomer; (**E**) GQ1e(d18:1/18:0) isomer.

HR MS screening demonstrated that the gangliosidome of macroalbuminuric patients is characterized by elevated overall sialic acid content. No less than four pentasialo gangliotetraoses of GQ1 class were identified in the A3 sample with a high mass accuracy. The ion at *m*/*z* 812.7068, detected only in the screening mass spectrum of A3, was assigned, according to mass calculation, to the triply deprotonated and sodiated GQ1(d18:1/18:0). In order to structurally characterize this species associated to macroalbuminuria in detail, we isolated the ion at *m*/*z* 812.7068 and submitted it to HCD MS/MS at HR in the negative ion mode. The targets of this tandem MS experiment were: (i) the investigation of the oligosaccharide chain structure; (ii) the confirmation of the ceramide composition, which was postulated considering the mass of the entire GQ1 molecules; (iii) collecting specific data upon the localization of the Neu5Ac monosaccharides along the oligosaccharide backbone, leading to isomer discrimination from the five most common GQ1(d18:1/18:0) structures (Figure 3) that might be present in the urine of macroalbuminuric patients.

**Figure 4:**
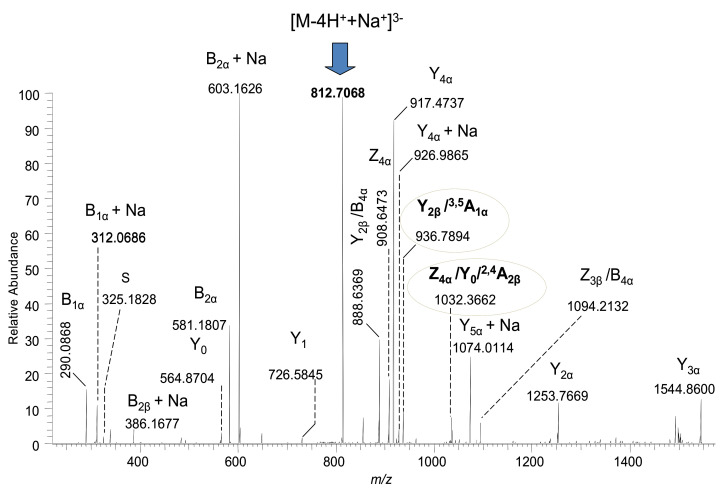
Structural analysis by (−) nanoESI HR HCD MS/MS of the [M-4H^+^+Na^+^]^3−^ precursor ion detected in the A3 sample at *m*/*z* 812.7068, which, according to mass calculation, corresponds to GQ1(d18:1/18:0). Acquisition time: 2 min; variable collision energies within 30–80 eV.

The fragmentation spectrum of the [M-4H+Na]^3−^ precursor ion detected at *m*/*z* 812.7068, generated by combining the TIC acquired for two minutes under variable collision energy within a 30–80 eV range, is depicted in Figure 4.

**Figure 5:**
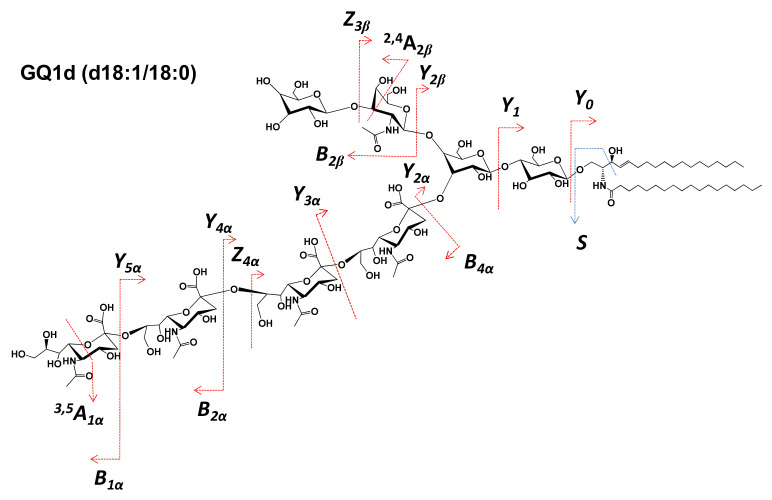
Scheme of the fragmentation by HCD MS/MS experienced by the [M-4H^+^+Na^+^]^3−^ precursor at *m*/*z* 812.7068 and the sequence ions diagnostic for the GQ1d structural isomer.

The MS/MS data validate the (d18:1/18:0) composition of the ceramide by the Y_0_ at *m*/*z* 564.8704 and Y_1_ at *m*/*z* 726.5845 corresponding to the Glc-Cer sequence, as well as the internal cleavage ion, S, at *m*/*z* 325.1828 corresponding to the C18:0 fatty acid (Figure 5). Likewise, the overall sialylation status of the molecule is also documented. Hence, by the B_1α_ ion detected at *m*/*z* 290.0868 and its sodiated counterpart at *m*/*z* 312.0686 corresponding to the detachment of one Neu5Ac residue from the parent ion, together with the disialo element detected at *m*/*z* 581.1807 and *m*/*z* 603.1626, confirm the occurrence of the Neu5Ac-Neu5Ac linkage.

Considering the most common linkage position of Neu5Ac residues at either the internal or external Gal, there are five possible structural candidates for GQ1(d18:1/18:0), as shown in Figure 3. However, the ion at *m*/*z* 1032.3662 formed by double bond and internal cross-ring cleavages and assigned to Z_4α_/Y_0_/^2,4^A_2β_, together with the ion detected at *m*/*z* 936.7894 as Y_2β_/^3,5^A_1α_, supports the attachment of all four Neu5Ac residues to the inner Gal. Although the incidence of other structural isomers cannot be completely excluded, these two fragment ions demonstrate that the structural motif consistent with the (D) candidate, which corresponds to the d isomer of GQ1(d18:1/18:0), is definitely present in the A3 sample. The scheme in Figure 5 depicts the fragmentation pathway experienced by the precursor ion corresponding to GQ1d(d18:1/18:0).

**Figure 6:**
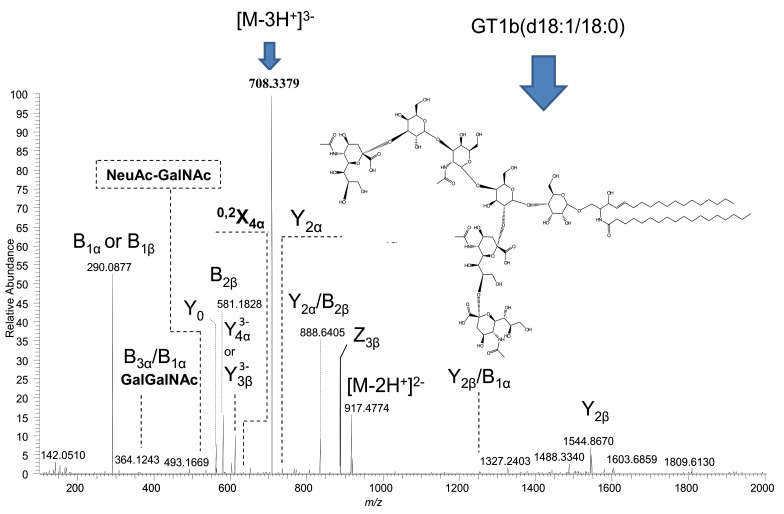
Structural analysis by (−) nanoESI HR HCD MS/MS of the [M-3H^+^]^3−^ precursor ion detected in the A3 sample at *m*/*z* 708.3379, which, according to mass calculation, corresponds to GT1(d18:1/18:0). Acquisition time: 2 min; variable collision energies within 30–80 eV. Inset: the structure of GT1b(d18:1/18:0) isomer deduced from the MS/MS data.

**Figure 7:**
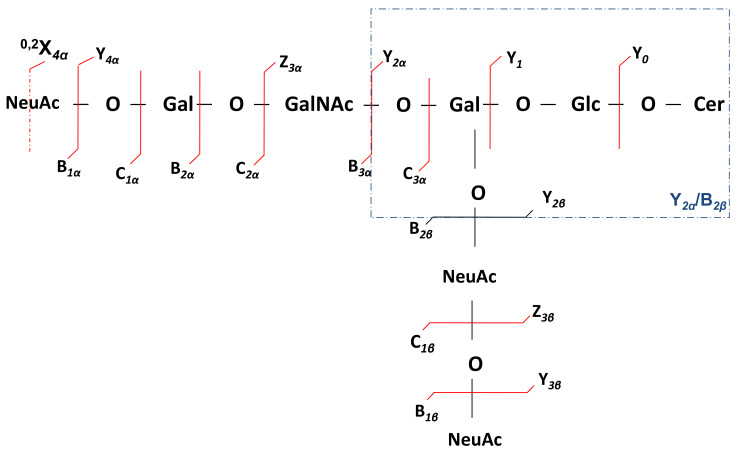
Scheme of the fragmentation experienced by the GT1 (d18:1/18:0) and the sequence ions diagnostic for the structure of GT1b isomer.

The next macroalbuminuria-associated polysialo candidate for detailed structural analysis is the [M-3H^+^]^3−^ ion detected solely in the screening mass spectrum of the A3 sample at *m*/*z* 708.3379 and assigned, on the basis of exact mass calculation, to GT1(d18:1/18:0). This ion was isolated and submitted to the fragmentation analysis by tandem MS using HCD. The product ion spectrum generated by summing up scans acquired for 2 min at variable collision energies between 30 and 80 eV is presented in Figure 6, together with the chemical structure of this species and the assignment of the major signals. The fragmentation mechanism under the employed MS/MS conditions, together with the diagnostic fragment ions, is depicted in Figure 7. To enhance the visibility of all assigned product ions, Table 3 complements the list of identified signals in the spectrum presented in Figure 6.

HCD fragmentation gave rise to a number of sequence ions, which occurred as a result of glycosidic bond and cross-ring cleavages. These ions are highly useful for the assignment of the entire carbohydrate sequence, with the identification of GT1 positional isomers, and for the confirmation of the ceramide aglycone composition.

The d18:1/18:0 type of ceramide is revealed by the Y_0_ ion at *m*/*z* 564.5353 and supported by the Glc-Cer sequence identified through the Y_1_ at *m*/*z* 726.5879. Furthermore, the ion assignment evidences the presence of a GT1α isoform. The ion at *m*/*z* 493.1669, with an NeuAc–GalNAc composition, may occur exclusively from GT1α, which is characterized by the sialylation of the GalNAc residue. The rest of the fragment ions suggest the existence of the GT1b structural isomer in the A3 sample. The structure of the GT1b is supported by all ions arising from the non-reducing end of the molecule, documented by the Y-type series, accompanied by two Z ions, of which one appears in lower abundance (Figure 6). However, the Y_2α_/B_2β_ detected as an ion of fair abundance at *m*/*z* 888.6405 gives evidence on the Gal–Glc–Cer sequence, possibly originating from either GT1a or GT1b isomer after the respective desialylation by either Neu5Ac or Neu5Ac-Neu5Ac detachment from the inner Gal or from an isomer containing a non-substituted inner Gal, such as the GT1d.

On the other hand, Y_2α_ detected at *m*/*z* 734.5701 substantiates the disialo Neu5Ac-Neu5Ac element localization at the inner Gal, a configuration consistent with the GT1b isomer. The presence of the disialo group is also evidenced by the B_2β_ ion at *m*/*z* 581.1828.

In the HCD MS/MS, a number of signals are related to the partial molecule desialylation, such as Y_4α_ and/or Y_3β_ ions corresponding to the disialylated Gg4Cer sequence; however, these ions can not indicate the attachment sites of Neu5Ac at the neutral Gg4 glycan core. Additionally, the signal at *m*/*z* 364.1243 corresponding to the Gal–GalNAc sequence assigned to the GT1b internal cleavage B_3α_/B_1α_ ion is attributable to the GT1c isomer because this glycoform contains a non-substituted terminal Gal–GalNAc disaccharide. Hence, except for the Y_2α_, which is associated to the GT1b, all ions could arise from other Neu5Ac positional isomers, such as GT1a, GT1α, GT1c, or even the rarely reported GT1d. On the other hand, Y_2α_ itself could be a result of a low-probability event, which also eliminated the Neu5Ac from the trisialo element linked to the inner Gal.

Given that, except for disclosing GT1α, the MS/MS data related to the glycosidic bond cleavages were unable to unambiguously assign other GT1 isomers in the A3 mixture, we more carefully analyzed the ring cleavage ions in order to discover possible inner fragmentation supporting GT1b. We noticed the low-intensity triply charged ion at *m*/*z* 634.6067, which is consistent with 0,2 type of cross-ring cleavage at one of the terminal sialic acid residues, which, in the case of GT1b species, can occur from either ^0,2^X_4α_ or ^0,2^X_3β_ or both. Because GT1b isomer is generally more common than GT1α, Figure 6 and Figure 7 present the ion assignment and the fragmentation scheme specific to GT1b.

Due to the Gal-GalNAc-Gal chain symmetry, there is no possibility of completely ruling out other structural isomers. Although the HCD MS/MS data demonstrate the existence of GQ1d(d18:1/18:0), GT1α(d18:1/18:0), and GT1b(d18:1/18:0) in the A3 sample, the possible occurrence of other GQ1 and GT1 isomers cannot be excluded. However, our findings indicate that three isomers—GQ1d(d18:1/18:0), GT1b(d18:1/18:0), and GT1α(d18:1/18:0) are definitely present in the A3 sample and may be further studied as molecular markers of macroalbuminuria.

## 3. Materials and Methods

### 3.1. Subject Enrollment Criteria

In order to develop and validate the method, a screening cohort of Type 2 DM patients and healthy control subjects was assessed in a cross-sectional pilot study. A total of 30 Type 2 DM patients attending the Outpatient Department of Nephrology and the Outpatient Department of Diabetes and Metabolic Diseases were divided into 3 groups according to urine albumin/creatinine ratio (UACR) as follows: 10 patients with normoalbuminuria (A1, defined as UACR < 30 mg/g), 10 with microalbuminuria (A2, UACR 30–300 mg/g), and 10 with macroalbuminuria (A3, UACR > 300 mg/g). Ten age- and gender-matched healthy control subjects (C) attending a general practitioner’s office for routine checkup without known history of renal diseases and without DM (excluded by a value of HbA1c ≤ 5.6%) were also enrolled in this study.

All patients included in the study had minimum 5-year duration of DM, stable renal function for at least 2 years, good blood pressure control (<130/80 mmHg), negative urinary sediment, and a negative urine culture. The exclusion criteria were represented by other causes of proteinuria (other glomerular diseases, neoplasia, or autoimmune diseases), hematuria, poor control of DM (HbA1c > 10%), liver diseases, and pregnant or lactating women. The patients had no indication for kidney biopsy (lack of hematuria and of other causes of proteinuria, no rapid decline in GFR).

### 3.2. Ethics Statement

The study was conducted in accordance with the Declaration of Helsinki, and the protocol was approved by the Ethics Committee in Research of the Institution (Board of Human Studies—“Victor Babes” University of Medicine and Pharmacy Timisoara, Nr. 15/12.09.2016; County Emergency Hospital, Nr. 100/23.11.2016). All patients and controls agreed to participate in the study by signing an informed consent form.

### 3.3. Laboratory Assessments

The urine gangliosidome of 30 Type 2 DM patients was investigated in a cross-sectional pilot study by a comparative assay with 10 healthy controls. Biochemical parameters referred to serum urea and creatinine, 24 h proteinuria, UACR, urine sediment, and urine culture. All patients were negative for urinary infections. The composition of native ganglioside mixtures was detected from 24 h collected urine samples. The urine specimens of patients and controls were short-term stored at −20 °C and thawed before assay. CKD was defined according to the KDIGO guideline for the evaluation and management of chronic kidney disease. The eGFR was calculated using the chronic kidney disease epidemiology collaboration equation formula (CKD-EPI creatinine 2009 equation) [16].

### 3.4. Ganglioside Extraction and Purification

Ganglioside extraction followed the method developed by Svennerholm and Fredman [17] and modified by Vukelić et al. [18]. We previously adapted this protocol for the extraction and purification of gangliosides from body fluids and applied it to cerebrospinal fluid (CSF) gangliosides. The method is described in detail in our previous study related to profiling and fragmentation analysis of CSF gangliosides by mass spectrometry [19]. Briefly, lipids were extracted twice using a chloroform (C)/methanol (M)/water (W) mixture to a total volume ratio of 1:2:0.75 C/M/W, with “W” corresponding to urine; therefore, the mixture contained 4 mL of C, 8 mL of M, and 3 mL of urine sample. Analytical-grade chloroform and methanol were purchased from Merck (Darmstadt, Germany) and used without further purification. After complete separation of the phases, following the procedures described in [19], the upper phase containing polar glycosphingolipids was collected.

Purification of the collected crude gangliosides was achieved in several steps [19]: the removal of precipitated protein–salt complexes, followed by centrifugation, gelfiltration on a Sephadex G-25 column (Sigma-Aldrich, Burlington, MA, USA) in order to remove low-molecular-weight contaminants, and finally, overnight dialysis at 4 °C against water. The gangliosides were extracted under identical conditions from all urine aliquots, yielding the A1 (normoalbuminuria), A2 (microalbuminuria), A3 (macroalbuminuria), and C (control) mixtures.

### 3.5. Sample Preparation for Mass Spectrometry

The purified A1, A2, A3, and C ganglioside extracts were evaporated to complete dryness in a SpeedVac Concentrator SPD 111 V system (Savant, Düsseldorf, Germany) coupled to a vacuum pump. For MS screening, each dry extract was dissolved in pure methanol to generate the stock solution andstored at −20 °C. Prior to MS analysis, the stock solutions were centrifuged in a Sigma 2–16 model centrifuge (Sartorius AG, Göttingen, Germany). For Orbitrap MS infusion, working samples with a concentration of 5 pmol·μL^−1^ in pure methanol were obtained by diluting aliquots from the stock solution. Ganglioside concentration in the infused solution was calculated for an average molecular weight of 2000 g mol^−1^.

### 3.6. Orbitrap Mass Spectrometry with nanoESI

The MS experiments were performed on an LTQ Orbitrap Velos Pro™ mass spectrometer (Thermo Fisher Scientific, Bremen, Germany) equipped with an offline nanoES source ES 259 (Thermo Fisher, Bremen, Germany).

The instrument is a hybrid mass spectrometer combining two different types of mass analyzers and providing a multitude of advantages: high sensitivity and accuracy, good quality of data and speed of analysis, potential for detection of minor components in complex mixtures, low sample consumption, and avoidance of cross examination and carryover from sample to sample.

The first analyzer is a dual-pressure linear quadrupole ion trap capable of isolating an ion with a specific mass-to-charge ratio (*m*/*z*) and of activating it by putting kinetic energy into the ion with the specific *m*/*z* in order to cause collision-induced dissociation. The second analyzer is the Orbitrap, which consists of a cylindrical outer electrode and a barrel inner electrode, trapping the ions in circular orbits around the inner electrode. It has excellent resolving power and offers the possibility of sequencing complex ionic species in multiple-stage MS (MS^n^) experiments by efficient fragmentation techniques.

Borosilicate capillaries (10 cm long) were pulled with a Sutter p-97 micropipette puller to produce electrospray capillaries with 10 µm tip sizes and taper lengths of 4 mm. A volume of 10 µL of the solution at a concentration of 5 pmol·μL^−1^ in methanol was introduced into the back of the emitter, and a 0.25 mm platinum wire was inserted into the solution. The potential values applied to the platinum wire and the cone were adjusted permanently to achieve an efficient ionization of the components. The nanoESI process was initiated by setting the instrumental parameters as follows: nanoESI voltage, 0.80 kV; cone voltage, 40–60 V; desolvation temperature, 80 °C; S-lens RF level, 60%. These values of the MS parameters enhanced the ionization and a steady sprayand, at the same time, minimized the in-source fragmentation of the labile Neu5Ac residue attached to the oligosaccharide core of the ganglioside molecule.

All mass spectra (MS and tandem MS) were acquired in HR mode, negative ion mode detection, and within the 200–2000 *m*/*z* range. MS scans were acquired with the resolution set to 60,000. The mass spectrometer was operated and controlled by LTQ Tune Plus v2.7 (Thermo Scientific, Bremen, Germany), and MS data acquisition and processing were achieved using Xcalibur 3.0.63 software (Thermo Scientific, Bremen, Germany).

MS/MS experiments were performed in the LTQ by CID in the collision cell using helium 5.0 purity at a pressure of 50 psi as the collisional gas. The MS/MS scans were acquired with resolution set to 20,000. Ion selection and fragmentation were performed manually. The precursor ions were selected within an isolation width of 1 *m*/*z* unit and fragmented by HCD using variable-collision energies within the 30–80 eV range to enhance coverage of fragment ions.

The TICs and mass spectra derived by combining the accumulated scans were processed using Xcalibur 2.1 software (Thermo Scientific, Waltham, MA, USA), which allows for the extraction of the spectra, as well as their smoothing and subtraction.

Prior to the experiments, the *m*/*z* scale was externally calibrated using the dedicated reference commercially known as Pierce^®^ ESI Negative Ion Solution from Thermo Scientific (Waltham, MA, USA). In negative ion mode, this standard provided a spectrum with a fair ionic coverage of the *m*/*z* range scanned in both MS and tandem MS experiments. Following the calibration procedure, the average mass accuracy was situated around 3 ppm.

For the optimization of the nanoESI MS and MS/MS conditions in negative ion mode, comparison, and data evaluation, the following standard ganglioside fractions, which are commercially available, were measured: GM1 (bovine brain); GD1a, GD1b, GT1b, and GQ1b (porcine brain);and GM3 and GD3 (bovine milk) from Avanti Polar Lipids (Birmingham, AL, USA), as well as the whole ganglioside extract from bovine brain, commercially known as Cronassial mixture, from Abano Terme (Padua, Italy).

### 3.7. Abbreviation of Gangliosides

For ganglioside assignment, the abbreviation system introduced in 1980 by Svennerholm [20], together with the recommendations from 1998 of IUPAC-IUB Commission on Biochemical Nomenclature, [21] was applied as follows:

LacCer-Galβ4Glcβ_1_Cer; GM3-II^3^-α-Neu5Ac-LacCer; GD3-II^3^-α-(Neu5Ac)_2_-LacCer; GT3-II^3^-α-(Neu5Ac)_3_-LacCer; GM2-II^3^-α-Neu5Ac-Gg_3_Cer; GD2-II^3^-α-(Neu5Ac)_2_-Gg_3_Cer; GM1a or GM1-II^3^-α-Neu5Ac-Gg_4_Cer; GM1b-IV^3^-α-Neu5Ac-Gg_4_Cer; GalNAc-GM1b-IV^3^-α-Neu5Ac-Gg_5_Cer; GD1a-IV^3^-α-Neu5Ac, II^3^-α-Neu5Ac-Gg_4_Cer; GD1b-II^3^-α-(Neu5Ac)_2_-Gg_4_Cer; GT1b-IV^3^-α-Neu5Ac, II^3^-α-(Neu5Ac)_2_-Gg_4_Cer; GQ1b-IV^3^-α-(Neu5Ac)_2_, II^3^-α-(Neu5Ac)_2_-Gg_4_Cer; nLM1 or 3′-nLM1-IV^3^-α-Neu5Ac-nLc_4_Cer; LM1 or 3′-isoLM1-IV^3^-α-Neu5Ac-Lc_4_Cer; nLD1-disialo-nLc_4_Cer.

### 3.8. MS Data Interpretation

Because no dedicated computer software, databases, or other IT services to assist the interpretation of the ganglioside screening and tandem mass spectra are available to date, in this study, the detected molecular ions were assigned to ganglioside species by exact mass calculation and on the basis of the information we had acquired previously on this type of glycosphingolipid and the known biosynthesis pathways. In the interpretation of the MS and MS/MS data and mass calculations, we were assisted by the large inventory of ganglioside molecular and fragment ions that we have identified and characterized in various human tissues and fluids to date using advanced MS approaches. These structures represent our original databases, which were included in our previously published studies [12,19,22,23,24,25,26,27,28,29,30,31]. The novel species reported in this work complete our currently existing ganglioside record.

The assignment of the oligosaccharide backbone sequence ions generated within the HCD MS/MS followed the generally accepted nomenclature [24,25].

## 4. Conclusions

In this study, we developed an approach based on HR MS and tandem MS for the determination of gangliosidome in DKD by a comparative assay of urinary extracts from normo-, micro-, and macroalbuminuric patients, as well as healthy controls, carried out under identical solution and instrumental conditions. The final goal of our study was to determine the changes occurring in the composition and structure of renal gangliosides expressed in DKD vs. controls and in different stages of the disease.

The high sensitivity, reproducibility, resolution, and mass accuracy provided by the MS platform optimized for this purpose allowed us to establish that the sialylation degree of the expressed species appears to play an important role in the progression of the disease, representing a marker of DKD, even in the normoalbuminuria stage of Type 2 DM patients. Additionally, the observed modifications in the composition of Cer show that, next to the glycan structure, the lipid moiety can also be considered a molecular fingerprint for DKD stages.

In the more advanced phase of the research, MS/MS by HCD was employed for the structural analysis of the species with a possible biomarker role. In the case of the A3 sample, the molecular ions, which, according to mass calculation, were found to correspond to GQ1(d18:1/18:0) and GT1(d18:1/18:0), respectively, were isolated and submitted to fragmentation in the collision cell of the instrument. The aim of these experiments was to collect information on the detailed structural configuration of the molecule. The optimized sequencing conditions, in particular the collision energy range and collisional gas pressure, induced specific cleavages of the glycosidic bonds, which resulted in the formation of fragment ions detected as relevant signals in the tandem mass spectra. A number of ions were found diagnostic for certain isomers related to the localization of the Neu5Ac moieties in the carbohydrate chain of the ganglioside molecule. Based on these structurally informative sequence ions, we discovered that GQ1d(d18:1/18:0), GT1α(d18:1/18:0), and GT1b(d18:1/18:0) isomers are present in the A3 sample and are associated to macroalbuminuria.

The obtained results require further validation of some hypotheses in longitudinal studies conducted on larger cohorts in order to prove a relation of causality between the complexity of the gangliosides detected in the urine of DKD patients and the very early renal involvement in the course of Type 2 DM, even in the normoalbuminuria stage. However, we consider that the present findings open a research direction towards the investigation of the role in disease progression played by sialylation, *O*-acetylation, and *O*-fucosylation of gangliosides, as well as their modifications by *O*-GalNAc and CH_3_COO^−^. Another beneficial aspect of these results is the possible development of procedures for early DKD diagnosis based on ganglioside profiling using advanced mass spectrometry.

## Figures and Tables

**Table 1:** Ganglioside species identified in A1, A3, and C samples by (-) nanoESI Orbitrap MS screening. d: dihydroxylated sphingoid base; t: trihydroxylated sphingoid base.

No.	Proposed Structure	*m*/*z*_theor_	Molecular Ion	*m*/*z*_exp_ A1	*m*/*z*_exp_ A3	*m*/*z*_exp_ C
1	GM2(d18:1/22:2)	477.6110	[M-3H^+^]^3−^	477.6100	-	-
2	GQ1(d18:1/16:0)	596.7751	[M-4H^+^]^4−^	-	-	596.7748
3	GQ1(d18:1/22:0)	617.7992	[M-4H^+^]^4−^	-	-	617.7979
4	GM3(d18:0/24:0)	645.3958	[M-H_2_O-4H^+^+2Na^+^]^2−^	-	645.3956	-
5	GP1(d18:1/18:0)	676.5571	[M-4H^+^]^4−^	676.5569	-	-
6	GT1(d18:0/16:0)	699.0040	[M-3H^+^]^3−^	-	-	699.0037
7	GT1(d18:1/18:0)	708.3382	[M-3H^+^]^3−^	-	708.3379	-
8	GT1(d18:1/18:1)	707.6681	[M-3H^+^]^3−^	707.6684	-	707.6684
9	GT1(t18:0/18:0)	714.3514	[M-3H^+^]^3−^	714.3513	-	714.3513
10	GT1(d18:1/20:0)	717.0221	[M-3H^+^]^3−^	-	-	717.0220
11	GT1(d18:0/20:0)	718.3636	[M-3H^+^]^3−^	-	718.3637	-
12	GT1(t18:0/20:0)	723.6952	[M-3H^+^]^3−^	723.6949	-	-
13	GD3(d18:1/18:1)	733.9043	[M-2H^+^]^2−^	733.9038	-	-
14	GD3(d18:1/18:0)	735.9199	[M-2H^+^]^2−^	735.9195	-	-
15	GT1(t18:1/24:3)	739.6953	[M-3H^+^]^3−^	-	739.6950	-
16	GD3(d18:1/20:1)	747.9199	[M-2H^+^]^2−^	-	747.9195	-
17	GM1(d18:1/16:1)	756.9070	[M-2H^+^]^2−^	-	756.9078	-
18	GQ1(d18:1/18:0)	812.7072	[M-4H^+^+Na^+^]^3−^	-	812.7068	-
19	GQ1(d18:1/20:0)	814.7253	[M-3H^+^]^3−^	814.7248	-	-
20	*O*-Ac GQ1(d18:1/18:0)	819.3803	[M-3H^+^]^3−^	-	819.3809	-
21	GQ1(d18:1/22:2)	830.0509	[M-4H^+^+Na^+^]^3−^	-	830.0504	-
22	GD2(d18:1/18:0)	847.4427	[M-3H^+^+Na^+^]^2−^	-	847.4421	-
23	GT3(d18:1/18:0)	891.4508	[M-3H^+^+Na^+^]^2−^	-	-	891.4503
24	GD1(d18:1/16:0)	903.4623	[M-2H^+^]^2−^		903.4616	903.4616
25	Fuc-GT3(t18:1/18:3)	969.4538	[M-3H^+^+Na^+^]^2−^	-	969.4534	-
26	GT2(d18:0/16:0)	979.9865	[M-3H^+^+Na^+^]^2−^	-	-	979.9861
27	Fuc-(CH_3_COO)GD1(d18:0/16:0)	1007.5125	[M-2H^+^]^2−^	-	-	1007.5118
28	GT1(d18:1/16:0)	1059.9997	[M-3H^+^+Na^+^]^2−^	-	1059.9988	-
29	GT1(d18:1/18:0)	1063.0263	[M-2H^+^]^2−^	-	1063.0260	-
30	GT1(d18:1/18:0)	1085.0084	[M-4H^+^+2Na^+^]^2−^	-	-	1085.0077
31	GT1(d18:1/22:0)	1091.0573	[M-2H^+^]^2-^	-	-	1091.0560
32	*O*-Ac GT1(d18:0/24:0)	1129.0721	[M-H_2_O-3H^+^+Na^+^]^2−^	1129.0652	1129.0649	-
33	GalNAc-GS1(t18:1/18:0)	1176.8277	[M-4H^+^+Na^+^]^3−^	-	1176.8266	-
34	GQ1(d18:1/18:0)	1219.5657	[M-3H^+^+Na^+^]^2−^	1219.5639	-	-
35	GQ1(d20:1/18:0)	1244.5711	[M-4H^+^+2Na^+^]^2−^	-	1244.5669	-
36	Fuc-GT1(d18:0/24:0)	1179.1103	[M-2H^+^]^2−^	-	1179.1094	-
37	GalNAc-GQ1(d18:1/18:0)	1310.1289	[M-2H^+^]^2−^	-	1310.1271	-

**Table 2:** Tabulated view of the identified urine ganglioside classes together with their differential expression in A1, A3, and C samples. Symbols: x = the class was detected; **-** = the class was not detected.

Ganglioside Class	A1	A3	Control
GM1	-	x	-
GM2	x	-	-
GM3	-	x	-
GD1	-	x	x
GD2	-	x	-
GD3	x	x	-
GT1	x	x	x
GT2	-	-	x
GT3	-	-	x
GQ1	x	x	x
GS1	-	x	-
GP1	x	-	-
*O*-Ac-modified species	x	x	-
Fuc-modified species	-	x	-
Fuc-CH_3_COO-modified species	-	-	x
GalNAc-modified species	-	x	-

**Table 3:** Assignment of the fragment ions detected in the negative ion MS/MS of the precursor molecular ion at *m*/*z* 708.3379 corresponding to GT1(d18:1/18:0) ganglioside marker in the A3 sample.

No.	Type of Fragment Ions	*m*/*z*
1.	Y_0_	564.5353
2.	Y_1_	726.5879
3.	C_3α_	673.2298
4.	C_2α_	470.1509
5.	Y_2α_	734.5701
6.	B_3α_	657.7908
7.	B_1β_ or B_1α_	290.0877
8.	Z_3β_(doubly deprotonated)	908.4721
9.	C_1β_ or C_1α_	308.2954
10.	B_2β_	581.1828
11.	Y_2α_/B_2β_	888.6405
12.	Y_2β_	1544.8670
13.	Z_3α_	1674.6733

## Data Availability

Not applicable.
